# IL-21 Receptor Expression in Human Tendinopathy

**DOI:** 10.1155/2014/481206

**Published:** 2014-03-13

**Authors:** Abigail L. Campbell, Nicola C. Smith, James H. Reilly, Shauna C. Kerr, William J. Leach, Umberto G. Fazzi, Brian P. Rooney, George A. C. Murrell, Neal L. Millar

**Affiliations:** ^1^Institute of Infection, Immunity and Inflammation, College of Medicine, Veterinary and Life Sciences University of Glasgow, 120 University Avenue, Glasgow G12 8TA, UK; ^2^Columbia University College of Physicians & Surgeons, New York, NY, USA; ^3^Department of Orthopaedic Surgery, Western Infirmary, Glasgow, UK; ^4^Department of Orthopaedic Surgery, Orthopaedic Research Institute, University of New South Wales, St George Hospital Campus, Sydney, NSW, Australia

## Abstract

The pathogenetic mechanisms underlying tendinopathy remain unclear, with much debate as to whether inflammation or degradation has the prominent role. Increasing evidence points toward an early inflammatory infiltrate and associated inflammatory cytokine production in human and animal models of tendon disease. The IL-21/IL-21R axis is a proinflammatory cytokine complex that has been associated with chronic inflammatory diseases including rheumatoid arthritis and inflammatory bowel disease. This project aimed to investigate the role and expression of the cytokine/receptor pair IL-21/IL-21R in human tendinopathy. We found significantly elevated expression of IL-21 receptor message and protein in human tendon samples but found no convincing evidence of the presence of IL-21 at message or protein level. The level of expression of IL-21R message/protein in human tenocytes was significantly upregulated by proinflammatory cytokines (TNF**α**/IL-1**β**) *in vitro*. These findings demonstrate that IL-21R is present in early human tendinopathy mainly expressed by tenocytes and macrophages. Despite a lack of IL-21 expression, these data again suggest that early tendinopathy has an inflammatory/cytokine phenotype, which may provide novel translational targets in the treatment of tendinopathy.

## 1. Introduction

Overuse tendon injuries, namely, tendinopathies, pose a significant clinical problem in musculoskeletal medicine [1]. The intrinsic pathogenetic mechanisms underlying the development of tendinopathies are largely unknown [2, 3]; however, several model systems have recently implicated proinflammatory cytokines [4–6]. The interaction between cytokine production and inflammation is critical to tissue homeostasis and plays a key role in many diseases including rheumatoid arthritis and cardiovascular disease. Previous studies highlight a huge influx of inflammatory cells, particularly mast cells [7, 8], at the site of tendon injury with the subsequent release of growth factors and cytokines [9, 10]. These factors chemotactically promote innate immune cell migration into the injured tissue and produce cytokines and growth factors (e.g., TGF-*β*, IGF-1, and basic FGF) in an attempt to produce tissue resolution [11, 12].

Tenocytes demonstrate endogenous expression of various cytokines such as TNF-*α*, IL-1*β*, IL-6, IL-10, VEGF, and TGF-*β* [13–15]. Increased amounts of IL-1*α*, IL-1*β*, TNF-*α*, and IFN-*γ* were present in inflamed native equine tendon [16], while inflammatory gene expression was altered in a rodent supraspinatus tendon overuse model [17]. Mechanical factors also influence tendon cytokine profile whereby cyclic strain has been shown to induce VEGF expression in tenocytes [18]. Conversely, stress deprivation leads to an overexpression of cytokines including IL-1*β* and TNF-*α* in the patellar tendon with subsequent mechanical deterioration of the tendon [19] with additional investigation in IL-6 deficient mice showing decreased tendon healing properties [5]. These results fit the pathogenetic theory that inflammation drives early tendinopathy, when microrupture of tendon induces expression of damage-associated molecular patterns (DAMPs) in an attempt to produce tissue healing [20].

IL-21 is a proinflammatory cytokine of the IL-1 family and is produced mainly by CD4+ lymphocytes and natural killer T cells (NK) [21]. It is known to modulate T-cell proliferation, B-cell differentiation, and cytotoxic properties of NK cells, as well as the antigen presenting and T-cell activating abilities of dendritic cells [22]. The IL-21 receptor is composed of a specific IL-21 receptor alpha chain and the common gamma chain receptor and has been found to be expressed at significantly higher mRNA levels and by immunohistochemical staining in biopsy samples from patients with systemic sclerosis and rheumatoid arthritis (RA), compared to controls [23, 24]. Furthermore, IL-21R is present in higher levels in synovial fibroblasts and macrophages in RA, activating fibroblasts independently of TNF-*α* and IL-1*β* [25].

The purpose of this study was to investigate the presence and role of IL- 21 and IL-21R in human tendinopathy and was borne out of preliminary studies in animal and human models of tendinopathy in which the proinflammatory cytokines were found in increased levels [9, 26].

## 2. Materials and Methods

### 2.1. Human Model of Tendinopathy

All procedures and protocols were approved by the South East Area Sydney Ethics Committee under ACEC no. 99/101 and NHS Greater Glasgow Research Ethics Committee (West Section). All patients gave informed consent as per the study protocol. Fifteen supraspinatus tendon samples were collected from patients with rotator cuff tears undergoing shoulder surgery (Table 1). The mean age of the rotator cuff ruptured patients was 54 years (range, 35–70 years) and the mean tear size was 2.5 cm^2^. Samples of the subscapularis tendon were also collected from the same patients. Patients were only included if there was no clinically detectable evidence of subscapularis tendinopathy on a preoperative MRI scan or macroscopic damage to the subscapularis tendon at the time of arthroscopy—by these criteria they represented a truly preclinical cohort. An independent control group was obtained comprising 10 samples of subscapularis tendon collected from patients undergoing arthroscopic surgery for shoulder stabilization without rotator cuff tears. The absence of rotator cuff tears was confirmed by arthroscopic examination. The mean age of the control group was 35 years (range, 20–41 years).

### 2.2. Tissue Collection and Preparation

Arthroscopic repair of the rotator cuff was carried out using the standard three-portal technique as described previously [27]. The cross-sectional size of the rotator cuff tear was estimated and recorded as described previously [28]. The subscapularis tendon was harvested arthroscopically from the superior border of the tendon 1 cm lateral to the glenoid labrum. The supraspinatus tendon was harvested from within 1.5 cm of the edge of the tear prior to surgical repair. For immunohistochemical staining the tissue samples were immediately fixed in 10% (v/v) formalin for 4 to 6 hours and then embedded in paraffin. Sections were cut to 5 *μ*m thickness using a Leica-LM microtome (Leica Microsystems, Germany) and placed onto Superfrost Ultra Plus glass slides (Gerhard Menzel, Germany). The paraffin was removed from the tissue sections with xylene, rehydrated in graded alcohol, and used for histological and immunohistochemical staining per previously established methodologies [29].

### 2.3. Immunohistochemistry

For immunohistochemical studies, samples were rehydrated through a graded alcohol/xylene series and endogenous peroxide activity was blocked using 0.5% H_2_O_2_ in methanol. Antigen retrieval was performed in 0.01 M citrate buffer at pH 6.0 for 30 minutes. Nonspecific binding was blocked using 2.5% horse serum and 2.5% human serum in TBST. Avidin/biotin block was carried out using avidin D for 15 minutes, followed by biotin for 15 minutes. Primary antibody, rabbit anti-human polyclonal IL-21 antibody (Lifespan Biosciences, Seattle, WA, USA), was then followed by biotinylated goat anti-rabbit IgG as the secondary antibody. Slides were then incubated with Vectastain ABC kit and staining was visualized using Chromogen ImmPACT DAB, counterstained with haematoxylin. For IL-21R staining, mouse anti-human IL-21R primary antibody was used followed by biotinylated horse anti-mouse secondary antibody and conjugated with tyramide (Perkin Elmer, Waltham, MA, USA) via HRP. Positive (human tonsil tissue, human rheumatoid arthritis tissue) and negative control specimens were included, in addition to the surgical specimens for each individual antibody staining technique. Omission of primary antibody and use of negative control isotypes confirmed the specificity of staining.

We applied a scoring system based on previous methods to quantify the immunohistochemical staining [8]. Five random high power fields (×400) were evaluated by three independent assessors (NLM, JHR, ALC). In each field the number of positive and negatively stained cells was counted and the percentage of positive cells was calculated giving the following semiquantitative grading, grade 0 = no staining, grade 1 ≤ 10% cells stained positive, 2 = 10–20% cells stained positive, and grade 3 ≥ 20% cells positive.

### 2.4. Tenocyte Culture

Human tendon derived cells were explanted from hamstring tendon tissue of 5 patients (age 18–30 years) undergoing hamstring tendon ACL reconstruction. Cultures were maintained at 37°C in a humidified atmosphere of 5% CO_2_ for 28 days. Cells were subcultured and trypsinized at subconfluency, Cells from the 3rd passage were used in normoxic conditions.

### 2.5. RNA Extraction and PCR

RNA was isolated from cells with Trizol followed by QIAgen RNEasy Kit (Qiagen, Crawley, UK) with additional DNase step. cDNA was prepared from mRNA with AffinityScript (Agilent Technologies, CA, USA) per manufacturer instructions. SYBR green real-time PCR was performed (Applied Biosystems, CA, USA) with ABI Prism 7900HT sequence detection system (AB) and all samples were normalized to GAPDH, the housekeeping gene.

### 2.6. Statistical Analysis

Significance was assessed with two-way paired Student's *t*-tests (RT-PCR), Mann-Whitney *U* tests (histological staining comparison), and Kruskal-Wallis one-way ANOVA (patient comparison data) using Graph Pad PRISM 5 software (La Jolla, CA, USA). Statistics reported on real-time PCR data represent the mean of each experiment repeated in triplicate and then the pooled mean of *n* = 3 or 5.

## 3. Results

### 3.1. Expression Levels of IL-21R in Human Tendinopathy

**Figure 1:**
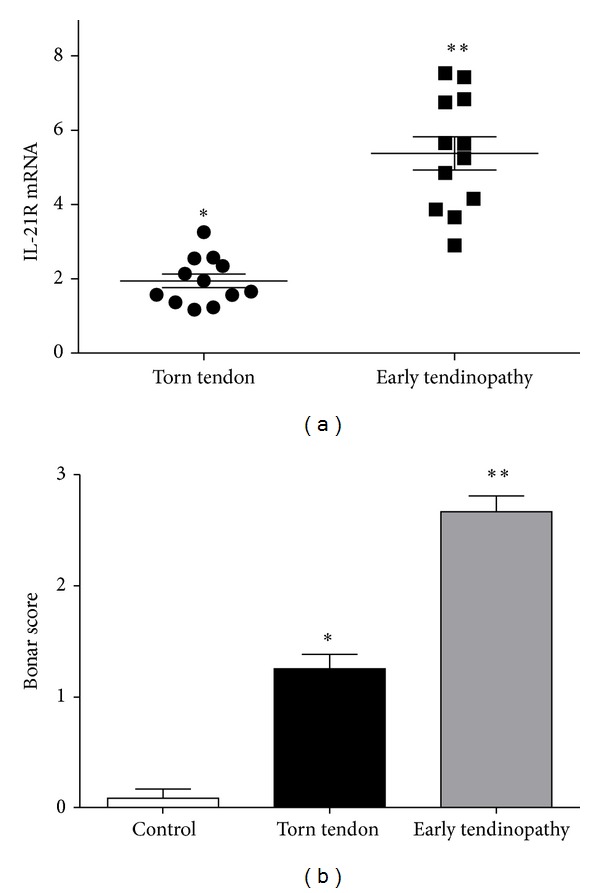
IL-21R message and protein in human tendinopathy. (a) The levels of mRNA (2^−ΔΔCT^) for IL-21R were determined by real-time PCR in torn tendon and early tendinopathy samples. Data are shown as the mean fold change ± SD of triplicate samples compared to control samples corrected to GAPDH and are representative of experiments using 12 individual donors of tendon tissue (*P < 0.05, **P < 0.01). (b) Semiquantitative scoring of IL-21R protein expression in tendon samples. Graphs illustrate relative expression of corresponding proteins in human tendon samples. Histological scoring system, 0 = no staining, 1 ≤ 10% cells positive, 2 = 10–20% cells positive, grade 3 ≥ 20% cells positive. Data displayed as mean ± SEM, *n* = 12 for supraspinatus and matched subscapularis, *n* = 10 for control group. (*P < 0.05, **P < 0.01).

IL-21R message and protein was significantly upregulated compared to control and torn tendon samples (Figure 1). In contrast to IL-21R, expression of IL-21 mRNA could not be detected in any tissue samples from the same tendon biopsy patients. There were no significant correlations between IL-21R expression and the mean duration of symptoms, patient age, or number of steroid injections.

**Figure 2:**
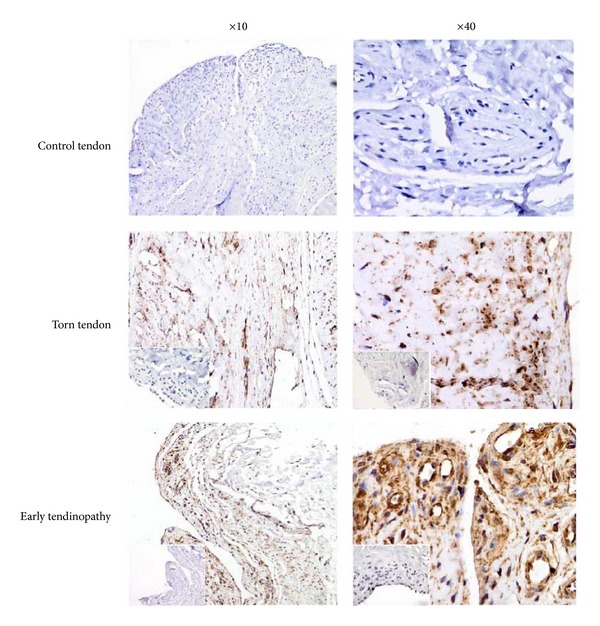
Immunohistochemical staining for IL-21R in human tendinopathy. IL-21R staining from control tendon, torn tendon, and early tendinopathy at ×10 and ×40 magnification stained for Isotype IgG in bottom left corner.

Torn tendon samples exhibited marked degeneration, mucoid change, and frank chondroid metaplasia (grade 4), whereas matched subscapularis tendon biopsies had grade-2-3 changes indicative of early tendinopathy as previously reported [26]. All control samples were classified as grade 1 consistent with normal fibrotendinous tissue with large distinct collagen fibrils. There were no significant correlations between Bonar score and the mean duration of symptoms or patient age. IL-21R was mainly expressed by cells in the sublining layer and by cells within the extracellular matrix in the early tendinopathy group (Figure 2). Within the torn group, only mild staining was noted in the sublining layer with minimal staining within the matrix. The majority of positive cells were tenocytes (confirmed with CD55 back to back staining) while further back to back staining with CD68 (macrophage) marker revealed a few (<10%) cells to be of macrophage lineage.

### 3.2. Expression of IL-21R by Tenocytes* In Vitro*

**Figure 3:**
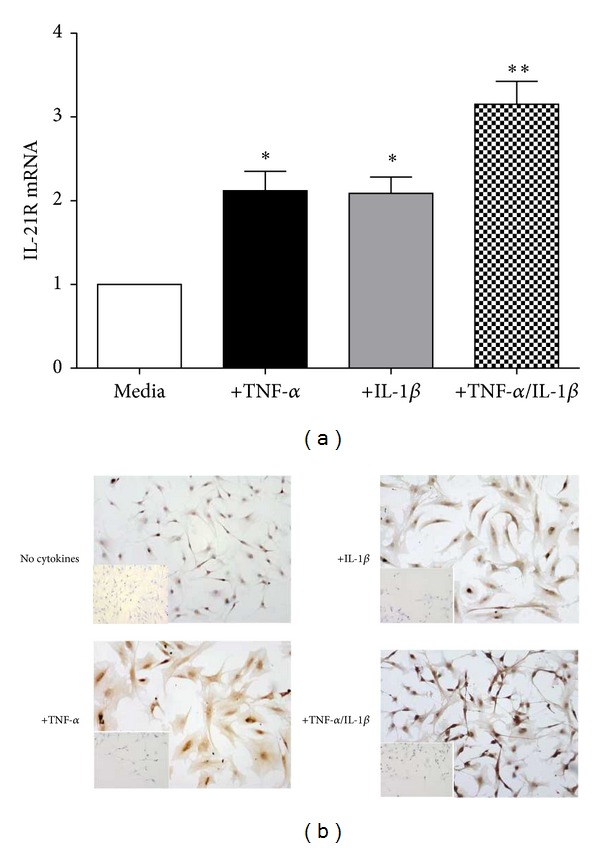
Upregulation of IL-21R in tenocytes* in vitro*. (a) The levels of mRNA (2^−ΔΔCT^) for IL-21R were determined by real-time PCR in explanted tenocytes* in vitro* following stimulation with IL-1, TNF, and in combination. Data are shown as the mean fold change ± SD of triplicate samples compared to no stimulated cells corrected to GAPDH and are representative of experiments using five individual donors of tendon explant tissue. (b) Immunostaining for IL-21R in explant tenocyte cultures stimulated as in (a) confirming changes at protein level.

IL-21R has been reported to be expressed in fibroblasts derived from synovial tissue [30]. Therefore, tendon fibroblasts were examined for their expression of IL-21 by IHC. Although no specific cell surface markers exist for tenocytes, we chose the surface marker CD55 that has been used by a collaborating laboratory (P.P.Tak) as a fibroblast marker to check assurance the cultured cells were indeed fibroblasts. IL-21R was detected in* in vitro* cultured tenocytes at both the message and protein level (Figure 3). Once again no expression of IL-21 message or protein was found in cultured tenocytes.

### 3.3. Upregulation of IL-21R by Proinflammatory Cytokines* In Vitro*

Next we examined whether IL-21R expression on tenocytes is regulated by proinflammatory cytokines. To this end, tenocytes were stimulated with IL-1*β* or TNF-*α*, two cytokines which are produced in excess in tendinopathy and known to regulate fibroblast activity. Both cytokines upregulated IL-21R expression and message (*P* < 0.01) and protein levels (Figure 3) with the most significant change seen in combination.

## 4. Discussion

Our study provides evidence that IL-21R is present in early tendinopathy and can be modulated in tenocytes by proinflammatory cytokines promoting the concept of IL-21R as inflammatory regulator in early tendon tissue damage.

Recent genome-wide association studies have provided convincing evidence that the chromosomal 4q27 region harboring the IL-2 and IL-21 genes is associated with chronic inflammatory disorders, including coeliac disease (a gluten-sensitive enteropathy), psoriasis, diabetes, rheumatoid arthritis, inflammatory bowel disease (IBD), asthma, and systemic lupus erythematosus (SLE) [31–33]. However, the precise role of the IL-21/IL-21R axis in fibroblasts and thus stromal cell pathologies remains to be established. IL-21R expression was found to be markedly increased in systemic sclerosis patients with skin biopsies showing keratinocytes as the predominant source of IL-21R [34]. IL-21R is also highly expressed in synovial macrophages and fibroblasts of rheumatoid arthritis (RA) patients [24]. T cells derived from the peripheral blood or synovial fluid of these patients produced high levels of Th1 cytokines after stimulation with IL-21, whereas blockade of IL-21 with the IL-21R/Fc fusion protein significantly inhibits inflammatory cytokine production in RA synovial membrane cultures [35]. Herein we, demonstrate significantly increased IL-21R expression in early tendinopathy similar to that reported in RA and SSc suggesting an “activated” IL-21R+ tenocyte phenotype to be involved in the inflammatory milieu of early tendon disease. This may contribute to tendon weakening in a similar manner to activated fibroblasts contributing to joint erosion in RA where it is has been shown that the IL-21/IL-21R axis is involved in dysregulated matrix metalloproteinase production [36].

More recently it has been demonstrated that IL-21 might also function as an important mediator of the crosstalk between immune and nonimmune cells, given that it can enhance the production of chemokines and tissue-degrading matrix metalloproteinases (MMPs) by epithelial cells [37] and fibroblasts [38]. Interestingly IL-21 was not detected by immunohistochemistry or real-time PCR which may suggest alternative ligands binding to IL-21R in tendinopathy. This theory has been proposed for RA as some receptors bind multiple cytokines; for instance, type II receptor complexes consisting of IL-4R*α* and IL-13R*α*1 heterodimers are activated by both IL-4 and IL-13 [39]. We have previously reported increased levels of IL-15 in early tendinopathy, which shares common cytokine receptor *γ*-chain (*γ*_*c*_), which is a functional component of the IL-21R complex [40]. Thus it may be that downstream activation of the IL-21R complex occurs in tendinopathy via IL-15 costimulation or alterative cytokine interactions. Secondly it may be that IL-21 expression occurs extremely early in the “tendinopathy spectrum” and that our cohort represents a later stage in the pathological process; however, we feel that the alternative ligand binding theory of IL-21R is a more likely scenario within tendinopathy pathology.

There are limitations inherent in our study. Firstly, as the control group was substantially younger than the patient group, age-related changes within the tendon samples could contribute to the degenerative picture and inflammatory cell expression seen in the matched subscapularis tendons. However, the lack of degenerative change on MRI and arthroscopic examinations suggests that the differences are truly at the cellular level as suggested by our work. Secondly, subscapularis tendon is functionally and organizationally distinct from supraspinatus and thus responds to mechanical loading in a different manner which may alter its cellular profile. Also control samples from subscapularis undergoing stabilization may not be truly “normal” controls but are currently the best available control tendon sample and this is reflected by a Bonar score of 0. It is reassuring, however, that we found the same inflammatory and vascular cell subtypes in matched subscapularis tissue indicating that the inflammatory response may be uniform within joints subjected to tendon degeneration. In addition having subscapularis samples from the same patient eliminates bias that may result from variation between individuals and has been previously shown to be useful method in sampling of tissues.

## 5. Conclusion

On the basis of these results we propose IL-21R as a potential inflammatory mediator in tendinopathy. Better understanding of the pathological cascade involving IL-21R could lead to the development of cell targeted treatment modalities in early human tendon disease.

## Figures and Tables

**Table 1:** Patient demographics and rotator cuff tear size.

Tear size	Control	Small (<1 cm^2^)	Medium (>1–3 cm^2^)	Large (>3–5 cm^2^)	Massive (>5 cm^2^)

Number of cases	10	6	6	3	3
Male : female ratio	8 : 2	4 : 2	3 : 3	2 : 1	1 : 2
Mean age in years (range)	35 (20–41)	55 (39–60)	58 (48–64)	54 (47–60)	66 (50–78)
Mean duration of symptoms in months (range)	6.2 (1–14)	8.1 (2–18)	6.8 (3–12)	9.8 (4–22)	6.9 (2–15)
Mean number of steroid injections	0	1.6	1.2	1.3	1.8

## Abbreviations

IL-21: Interleukin 21
IL-21R: Interleukin 21 receptor
TNF-*α*: Tumour Necrosis Factor alpha
IL-1*β*: Interleukin 1 beta
MRI: Magnetic resonance imaging.
